# Assessing the severity of medication administration errors identified in an observational study using a valid and reliable method

**DOI:** 10.1186/s40545-023-00653-x

**Published:** 2023-11-14

**Authors:** Lindemberg Assunção-Costa, Charleston Ribeiro Pinto, Juliana Ferreira Fernandes Machado, Cleidenete Gomes Valli, Luis Eugenio Portela Fernandes de Souza

**Affiliations:** 1 Department of Medicine, School of Pharmacy, Federal University of Bahia and National Institute for Phamaceutical Assistence and Pharmacoeconomy - INAFF, Rua Barão de Jeremoabo, 147, 2º andar, Campus Ondina, Salvador, Bahia 40170-115 Brazil; 2 Department of Medicine, School of Pharmacy, Federal University of Bahia, Salvador, Bahia Brazil; 3 National Institute for Pharmaceutical Assistance and Pharmacoeconomics - INAFF, Salvador, Bahia Brazil; 4 Institute of Collective Health, Federal University of Bahia, Salvador, Bahia Brazil

**Keywords:** Medication errors, Medication administration errors, Severity, Patient safety, Hospital, Observational

## Abstract

**Background:**

Epidemiological data on medication errors severity are scarce. The assessment of the prevalence and severity of medication errors may be limited because of several reasons, including a lack of standardization in the method of identifying medication administration errors and little knowledge about the appropriate assessment tools to measure severity. Thus, in this study, we aim to assess the potential severity of errors identified by direct observation in a teaching hospital.

**Methods:**

We used a validated method for assessing the potential severity of medication administration errors. Responses are scored on a 10-point scale. The 203 errors identified in a previous study were organized per similarity, resulting in 67 errors. This list was assessed by a panel of a physician, a nurse, and two pharmacists. The average score for each of the 67 errors was estimated considering the scores given by the 4 judges. Errors with a severity index < 3, between 3 and 7, and > 7 were considered minor, moderate, and severe, respectively.

**Results:**

Professionals classified the potential clinical significance of the errors as minor, moderate, and severe in 8.8% (18/203), 82.8% (168/203), and 8.4% (17/203) of the cases, respectively. Most errors considered potentially serious (41%, 7/17) were technical errors. Most potentially serious errors involved insulin. Regarding the administration route, nine (53%) potentially serious errors involved medications administered intravenously.

**Conclusions:**

Most of the errors were considered as potentially moderated by the expert panel; however, the frequency of potentially serious errors was higher than that in previous studies using the same methodology, which highlights the need for strategies to reduce their occurrence.

**Supplementary Information:**

The online version contains supplementary material available at 10.1186/s40545-023-00653-x.

## Background

Unsafe medication use and medication errors are among the leading causes of preventable harm in health systems worldwide [1]. In 2017, the World Health Organization launched the 3rd Global Patient Safety Challenge with an aim to strengthen these systems and reduce medication errors and preventable harms associated with the occurrence of these errors [2]. The magnitude and nature of harms associated with medication errors differ among low-, middle-, and high-income countries. However, epidemiological data on the occurrence of medication errors and their severity in many countries, including Latin American countries, are scarce [2–5]. Factors that may limit the assessment of the prevalence and severity of medication errors include differences in the definition of what constitutes medication use-related harm, limitations in the ability to define whether medication use-related harm is potential or real, and doubts about the tools used to assess the severity of harm (potential or real) and their use [6–8]. The term "severity of error" is typically used to describe the extent of the potential or real impact of medication errors. However, it does not refer to the error as such, but rather to the potential or real harm to the patient believed to be associated with the error. This distinction is important, given the obvious severity of the real "harm" to the patient compared with the potential of causing that harm. Furthermore, many errors are intercepted before they reach the patient and those that cause harm account for a small proportion of all errors [1, 8].

The assessment of potential and real harms involves two distinct processes: identification of the potential or real harm to the patient associated with a medication error and classification of error severity [9]. A potential harm is evaluated according to its expected severity. To evaluate a real harm, the severity of the harm is considered after the occurrence of a medication error [8, 9]. A recent systematic review conducted by Assunção-Costa et al. [5] identified ten studies in which the direct observation technique was used to assess the prevalence of medication administration errors (MAEs) in Latin American hospitals. None of these studies involved the use of tools to assess the severity of the errors that occurred, highlighting the need for a better understanding of the harms associated with MAEs in this region, as a strategy to reduce their occurrence and promote safe medicine use. A variety of tools are available for measuring and classifying harms associated with medication errors. In this study, we used the ten-point scale developed by Dean and Barber [10] to classify potential harms. This tool has been proven highly reliable and valid in studies conducted in the United Kingdom and Germany [11] and was recently validated for application in Brazil [12]. To our knowledge, this is the first study involving the use of a validated and reliable method of scoring the severity of MAEs in Brazil. This work is part of a larger study on MAEs in a university hospital and aims to assess the potential severity of the MAEs detected in this hospital.

## Methods

### Study design and location

A prospective observational study was carried out based on the disguised direct observation^[69]^ of medication administration errors in a public university hospital^[70]^ by Assunção-Costa et al. [6]. This study was conducted in two wards: a medical (21 beds) and a surgical (23 beds) clinic; both units have patients with more than one chronic disease, who use prescribed drugs from several pharmacological groups. The medical unit admits patients from neurology, neurosurgery, and orthopedics specialties. In contrast, the surgical unit admits female patients from gynecology, plastic surgery, urology, and otorhinolaryngology specialties.

In the nursing care routine of this hospital, nursing technicians are responsible for both the preparation and administration of medications, except chemotherapy drugs, and for bathing, feeding, and providing basic care to patients. In turn, the nurses are responsible for supervising the technicians, performing administrative duties, and applying bandages and catheters, among other duties.

### Assessment methodology

In this study, we used a scale validated for application in Brazil by AssunçãoCosta et al. [12] to measure the potential severity of medication erros [10, 13]; which confirmed an assessment by at least three professionals, regardless of their profession (a doctor, nurse, and pharmacist), is required to consider the rating scale reliable. In this scale, 0 represents no harm to the patient and 10 represents patient death.

Two hundred and three MAEs were identified in this study and categorized by type: time error, technique error, dose error, route of administration error, omission, extra dose, non-prescribed dose, and wrong pharmaceutical form. The definition of each type of error is described in Additional file 4. Subsequently, the errors were organized according to similarity by type of error by the researcher JFFM and verified by the principal investigator LAC, which resulted in a list of 67 errors (Additional file 1).

These errors were assessed according to their severity, replicating the assigned severity for the remaining 136 of the total 203 errors. This analysis was performed to make the assessment process objective and reduce the workload for the judges.

## Judges

Four professionals were selected (a physician, a nurse, and two pharmacists), who were working in the hospital area and had more than 3 years of experience in clinical settings. All the professionals received a file with the description of the 67 errors and the severity scale (Additional file 1) for evaluation. A letter with instructions on how to assess the potential severity of the errors was also sent (Additional file 2).

## Ethics approval statement

This study was submitted to the evaluation of the Research Ethics Committee of the Complexo Hospitalar Universitário Professor Edgard Santos and approved under opinion number 3.102.570/2019.

## Data analysis

The judges were asked to record their observations and the time required to assess all errors. An average of the scores assigned by the four judges was calculated and used as the severity index for each MAE. Errors with a severity index of < 3, 3 to 7, and between 7 and 10 were classified as minor, moderate, and severe, respectively, according to previous studies [10, 12].

## Results

According to the average score assigned by the judges for the potential clinical significance of the errors, 8.8% (18) were classified as minor, 82.8% (168) as moderate and 8.4% (17) as severe.

The average potential severity score was 5.2 (minimum score: 2.6, maximum score: 7.7; SD 1.2). The two pharmacists took 40 and 52 min, each, to assess the 67 cases, while the physician and the nurse took 48 and 62 min, respectively (average time required: 50 min). The scores and severity levels assigned for each case are listed in Additional file 3. Table 1 describes some examples of errors classified as potentially minor, moderate, and severe. The case in which an observer had to intervene was classified as potentially severe (severity index: > 7).

**Table 1:** Examples of medication errors by severity index

MINOR (Severity index between 0 and 3)
An excess amount of Hyabak® (ophthalmic solution, 0.15%) was administered into the patient's right eye
The patient was prescribed dimethicone (drops, 75 mg/mL) but was administered one tablet (40 mg)
MODERATE (Severity index between 3 and 7)
Patient was on spironolactone (tablet, 100 mg). However, the medication was not administered
The patient was prescribed furosemide (solution for injection, 10 mg/mL). The dilution manual advises infusion between 1 and 2 min; however, administration was performed in 11 s
SEVERE (Severity index higher than 7)
Warfarin* (tablet, 5 mg) was offered to the patient; however, the medication was prescribed for another patient. Intervention was performed before administration
Patient on hydralazine (tablet, 25 mg). There was an instruction not to administer the antihypertensive on hemodialysis days. However, the drug was administered on the same day as hemodialysis

*Only case that required interventions to avoid MAEs

Most of the errors classified as potentially severe (41%, 7 errors) were technical errors. Dose, omission, and overdose errors were also classified as potentially severe, with a frequency of 18% each (three errors of each type), in addition to one non-prescribed dose error. According to the Anatomical Therapeutic Chemical categorization, potentially severe errors involved medicines in category A—digestive tract and metabolism (29%); J—anti-infectives for systemic use (29%); B—blood and hematopoietic organs (24%); and R—the respiratory system, C—the cardiovascular system, and N—the nervous system (6% each). The medicine associated with most of the potentially severe errors was insulin (two dose and omission errors, each, and one technical error). Regarding the route of administration, nine (53%), five (29%), and three (18%) potentially serious errors were, respectively, associated with intravenous, subcutaneous, and oral medication administration.

## Discussion

In this study, we assessed the potential severity of MAEs identified by direct observation in a Brazilian university hospital. The frequency of errors classified as potentially moderate and severe in our study was higher than that in international studies [3, 6, 10, 13–15]. The average severity score for MAEs was also higher (5.2) than those reported by Dean and Barber [10] (2.7) and Taxis and Barber [13] (3.1). We do not know the reasons why both the frequency of severe errors and the average severity score were high in our study. However, we do know that some related factors may have contributed to these findings, such as the intravenous route of administration and use of potentially dangerous medications, which are already known to cause the greatest harm to patients when an error occurs [5, 16, 17].

A study conducted in a Brazilian university hospital found a greater association between the intravenous administration route and the occurrence of errors, especially in the medication preparation phase [18]. The relationship between the severity of potential errors and the intravenous administration route is well established. A similar study involving a sample of 10 wards from two hospitals in England showed that errors in intravenous administration occurred in half of the doses administered and caused potential harm in one-third of the cases [19]. Another similar study was conducted in Germany, in which the same author found that 3% of 65 MAEs associated with the intravenous route of administration were severe. Several international studies have demonstrated the high degree of severity of errors associated with intravenous administration [15, 17, 19, 20]. However, there is no information on the severity of MAEs in Latin America, especially Brazil. Thus, it is necessary to better investigate the potential for harms caused by medication errors to patients, especially in low- and middle-income countries [3, 5, 15, 21].

Another important finding was that almost half of the errors assessed as potentially severe were technical errors, which differs from the results found in national and international literature [5, 14, 15]. Furthermore, the potentially severe errors involved categories A, J, and B medications, with insulin being the main medication. The literature indicates that medication errors related to insulin are common, and approximately one-third of these cases involve fatal errors. Nguyen et al. [22]. examined insulin administration and found that most errors were potentially moderate and severe, emphasizing the need for interventions focused on clinically important errors because insulin requires timely dosing, administration, and careful monitoring (Additional file 5).

When studying the incidence or prevalence of medication errors, it is important to determine their clinical significance. However, it is often difficult to do so, because in many studies, the actual clinical outcomes are unknown due to the lack of longitudinal follow-up of patients or researchers intervening to prevent errors from causing harm to patients. There are several methods for assessing error severity. The two most common are the National Coordinating Council for Medication Error Reporting and Prevention severity index and the Dean and Barber method [10]. This was the first study using a validated scale to assess the severity of errors in Brazil [12]. The scale developed by Dean and Barber^10^ seems to be more suitable for use in research [23]. Assessment of potential error severity is complex and can be influenced by many factors. The use of this scale in future research may help determine the clinical significance of medication errors more clearly in the Brazilian context, contributing to the development of interventions aimed at reducing the associated harm. To the best of our knowledge, this is the first study on MAE assessment that has been conducted using a validated, reliable scale for potential error severity in Brazil.

## Conclusion

Most of the errors were considered as potentially moderated by the expert panel; however, the frequency of potentially serious errors was higher than that in previous studies using the same methodology, which highlights the need for strategies to reduce their occurrence.

### Supplementary Information

**Additional file 1. **List of 67 similar Medication Administration Errors.

**Additional file 2. **Guidance letter to judges.

**Additional file 3. **Scores and severity levels assigned for each observed MAE.

**Additional file 4. **Categories of Medication Errors.

**Additional file 5. **Grouping MAE.

## Data Availability

All data generated or analysed during this study are included in this published article [and its supplementary information files]. In case of further queries, the corresponding author may be contacted by email upon reasonable request.

## Abbreviations

MAE: Medication administration error
MAEs: Medication administration errors
SD: Standard deviation
