# Supplementary material for: Assessing the severity of medication administration errors identified in an observational study using a valid and reliable method
Source: J Pharm Policy Pract. 2023 Nov 14;16:143. doi: 10.1186/s40545-023-00653-x (PMC10648330; doi:10.1186/s40545-023-00653-x)
Supplement: Supplementary file 1 — Additional file 1. List of 67 similar Medication Administration Errors. [file 40545_2023_653_MOESM1_ESM.docx]

**Additional file 1–** List of 67 similar Medication Administration Errors

| 1 | Patient with systemic arterial hypertension, on continuous use of losartan 50 mg. The nursing technician did not administer the medication because he thought that the patient's blood pressure was low, and it would be referred to the Operating Room in the following hours. | | | 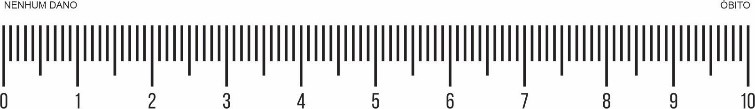 | | | |  |  |  |
| --- | --- | --- | --- | --- | --- | --- | --- | --- | --- | --- |
| 2 | Patient using calcium carbonate + vitamin D tablet (1250 mg + 400 IU). The drug was not administered but was checked in the prescription as being given. | | | 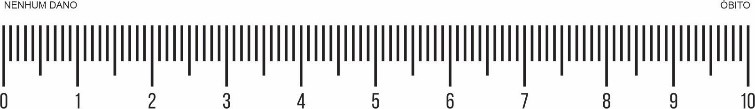 | | | |  |  |  |
| 3 | Prescribed potassium permanganate solution (1:80.000) for washing of skin lesions of the patient. The nursing technician records that she does not have the drug in the unit, but the drug was available. | | | 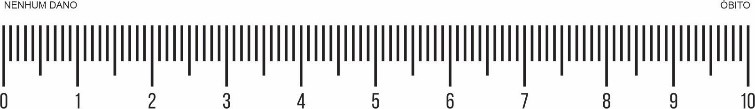 | | | |  |  |  |
| 4 | Patient on continuous use of dipyrone solution for injection 1g and codeine + paracetamol, both prescribed for pain control. Dipyrone was not prepared by the technique, because during the administration of codeine + paracetamol, the patient reported not feeling pain and technique did not administer dipyrone. Was  marked in the prescription as administered. | | | 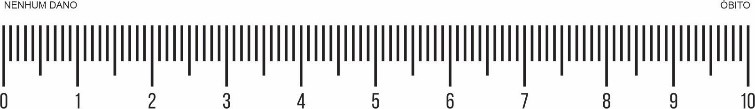 | | | |  |  |  |
| 5 | Patient on continuous use of dipyrone solution for injection 2g for pain control. It was checked in the prescription of one patient; however the drug was administered in another patient. | | | 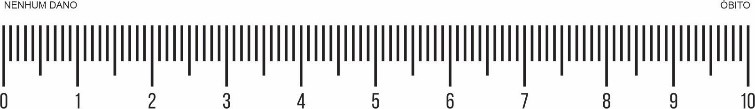 | | | |  |  |  |
| 6 | Patient using regular insulin. Prescribed 4 units if HGT between 190 and 250 mg/dL. Capillary glycemia (HGT) was 209 mg/dL. The administration not carried out. | | | 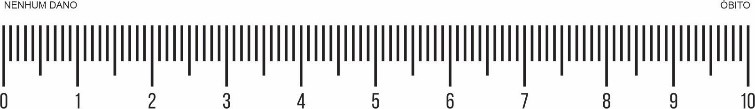 | | | |  |  |  |
| 7 | A patient using bromopride (ampoule 5 mg), complaining of pain in venous access, had interrupted administration. In the prescription was marked administered with observation of no access and administration of the drug was not completed. | | | 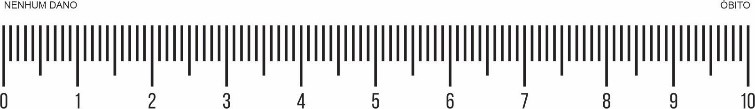 | | | |  |  |  |
| 8 | Patient using NPH Insulin (10 units). A patient registered in the prescription that did not administer medication because a patient had a risk of hypoglycemia. However, it had no information to refer to this decision. | | | 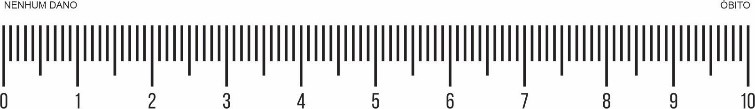 | | | |  |  |  |
| 9 | Patient using amitriptyline 25 mg. The drug was not administered and was found in the patient's box without justification. | | | 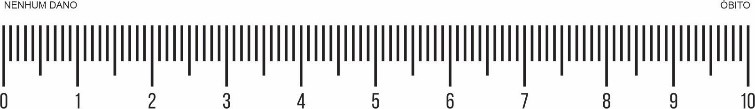 | | | |  |  |  |
| 10 | The patient was given bisacodyl (5 mg tablet) at 16:08 hours. At 16:36, an intervention was performed because the prescription guided the administration of four tablets to prepare for colonoscopy. | | | | | 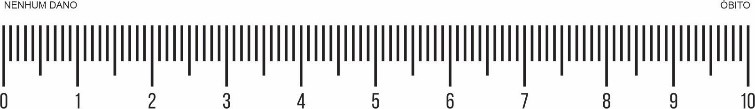 | | |  |  |
| 11 | Ivermectin 18mg has been prescribed and 12mg administered. | | | | | 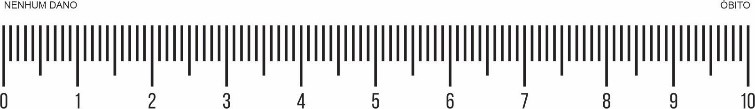 | | |  |  |
| 12 | Amitriptyline (02 tablets of 25 mg) has been prescribed. 1 tablet (25 mg) was administered. Patient reported that dose was wrong, technique was to check prescription and did not return. | | | | | 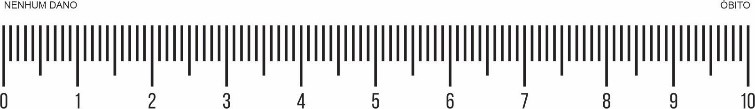 | | |  |  |
| 13 | Saline solution 0.9% (500ml) + glucose 25% (40 mL) was prescribed. Aspirated four glucose ampoules 25% and added to the 500 ml serum. Serum volume was not adjusted with the addition of 40 ml volume. Medicine time of 20 drops/minute. | | | | | 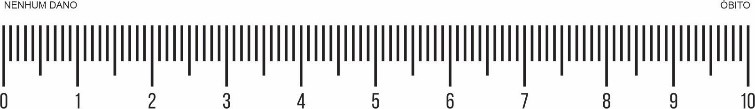 | | |  |  |
| 14 | Hyoscin + dipyrone 3ml has been prescribed. Administered 2 ml. | | | | | 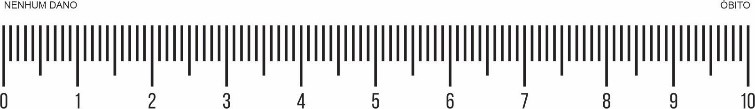 | | |  |  |
| 15 | Enoxaparin 70 mg, solution for injection, has been prescribed. A 60 mg + 10 mg syringe was used from a 20mg syringe (half of the contents with the syringe face down are discarded). The 20 mg syringe is not graded, and it is not possible to specify the final volume after being discarded half  of the content. | | | | | 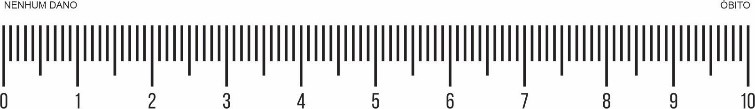 | | |  |  |
| 16 | Clindamycin (solution for injection 150 mg/ml, 4 ml) has been prescribed. The nursing technician disregarded some volume of the drug to remove air bubble from the equipment. Used pre-used equipment. It was not possible to specify the despised volume. | | | | | 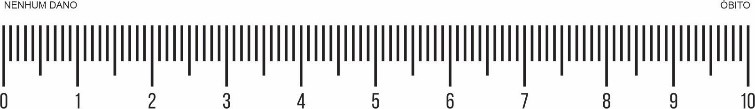 | | |  |  |
| 17 | It was prescribed 01 vial Ringer Lactate + 12.5 ml KCl + 7 ml NaCl + 10 ml glucose + 2.5 ml MgSo4. The technique brushed equipment after introducing electrolytes and had loss of volume of the drug that could not be specified quantity. 32mL of medicine was added, but the total volume after loss is not known. Flow programmed according to the  prescription. | | | | | 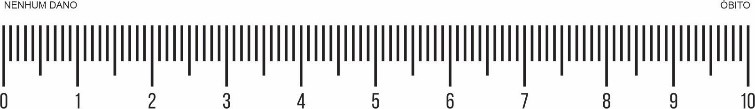 | | |  |  |
| 18 | Azathioprine 150mg (03 tablets) was prescribed, made 50 mg (01 tablet) | | | | | 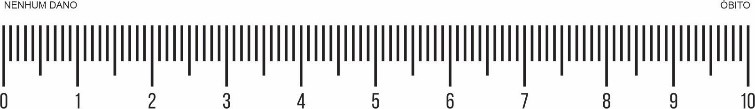 | | |  |  |
| 19 | Sulfamethoxazole + trimethoprim tablets (400 mg + 80 mg) were prescribed. Given 1 tablet. | | | | | 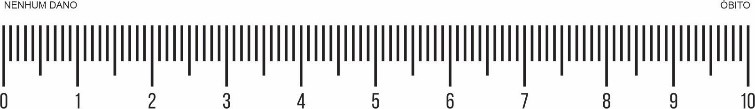 | | |  |  |
| 20 | It was prescribed 50mg of dimenhydrinate but administered 30mg (1 ampoule). | | | | | 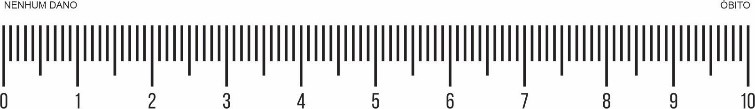 | | |  |  |
| 21 | Prescribed Hyabak® ophthalmic solution 0.15% (01 drop in each eye). Two drops were administered to the right eye. | | | | 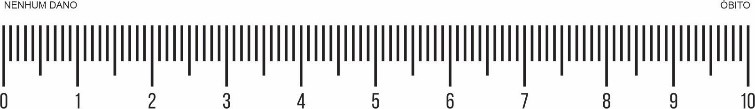 | | |  |  |  |
| 22 | Prescribed tramadol if necessary (solution for injection 50 mg/ml, 2 ml). Tramadol was diluted in a compatible solution, but they discarded part of the solution with the drug, and it was not possible to specify the impact of the loss; | | | | 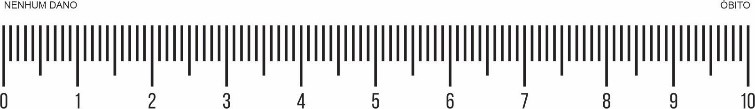 | | |  |  |  |
| 23 | Atenolol 2 tablets (50mg) have been prescribed. Administered 1 tablet (25mg); | | | | 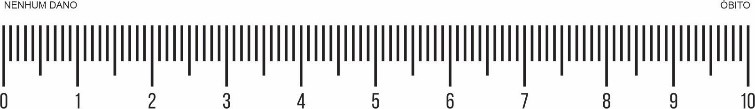 | | |  |  |  |
| 24 | Ibuprofen (oral suspension drops, 50 mg/ml) has been prescribed. The nursing technique did not position the dropper stake in the vertical position and there was no drop formation. The administered dose was incorrect; | | | | 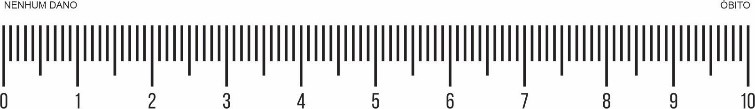 | | |  |  |  |
| 26 | Codeine 30mg was prescribed without association. Administered codeine 30mg + paracetamol 500mg. | | | | 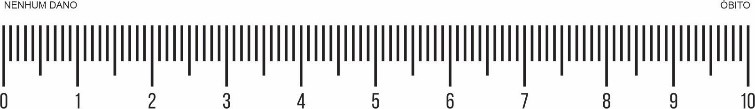 | | |  |  |  |
| 27 | Codeine (1 tablet 30 mg) was prescribed if pain, but medicine was administered continuously. | | | | 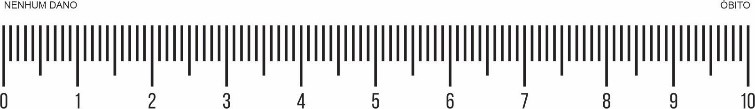 | | |  |  |  |
| 28 | Hydralazine (25 mg tablet) was prescribed, but there was an observation in the prescription not to do antihypertensive drugs on hemodialysis days (Tuesday, Thursday and Saturday). It was still administered on Thursday. | | | | 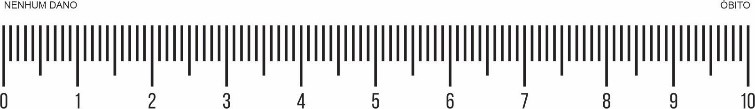 | | |  |  |  |
| 29 | Oral metoclopramide was prescribed, and an ampoule (5 mg/ml) administered intravenously for 23 seconds. | | | | 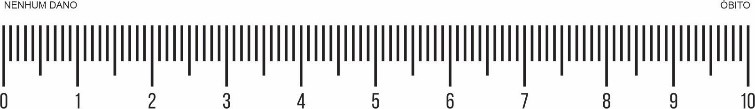 | | |  |  |  |
| 30 | Simple hyoscin (10mg) was prescribed orally. It was done intravenously in a time of 35 seconds. | | | | 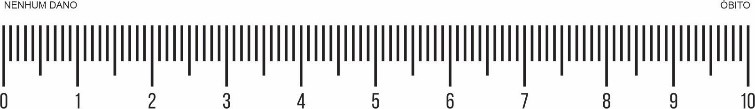 | | |  |  |  |
| 31 | The medicine dimenhydrinate + vitamin B has been prescribed, if necessary, orally. Intravenous solution (30 mg/ml) was administered. | | | | 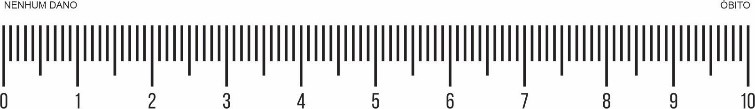 | | |  |  |  |
| 32 | Dimethicone oral emulsion (drops) was prescribed, and a 40 mg tablet was administered. | | | | 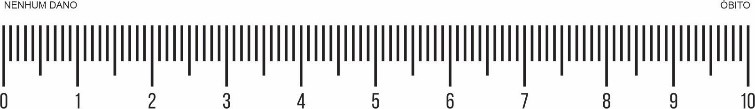 | | |  |  |  |
| 33 | Ondansentrona (1 ampoule of 4 mg) was prescribed. Dilution manual indicates infusion between 15-30 minutes. Done in an hour and 40 minutes. | | | | 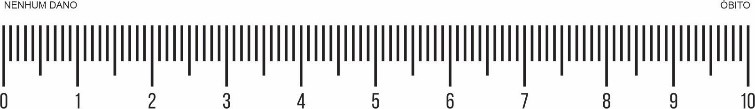 | | |  |  |  |
| 34 | Albumin (20%) 50 ml vials were prescribed. Administration occurred as follows: 1st and 2nd vials in 30 min (15 min each); 3rd vial in 35 min; 4th to 7th vial in 1:45h (approximately 25 min each). The dilution manual guides from 1 to 2 ml/min which corresponds to the minimum time of 25 minutes for each vial. Brushed equipment aspiring due to difficulty in fluidity. The contents left in the syringe were administered direct IV (about 5 ml). | | | | | 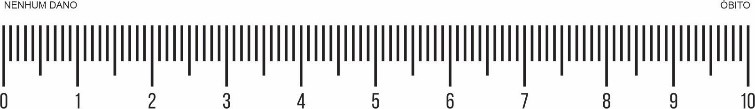 | | | |  |
| 35 | Prescribed meropenem (02 vials of 500 mg). the dilution manual guides infuse between 15-30 minutes, done in one hour. | | | | | 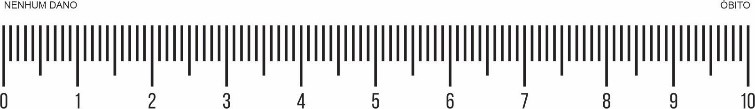 | | | |  |
| 36 | Prescribed furosemide (10 mg/ml – 2 ml), 2 ampoules. The dilution manual guides infuse between 1 and 2 min, done between 10-11 seconds. | | | | | 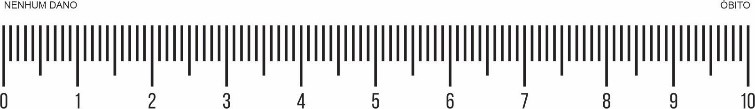 | | | |  |
| 37 | Saline solution 0.9% (500 ml vial) was prescribed for administration in 8 hours. Done in about four hours. | | | | | 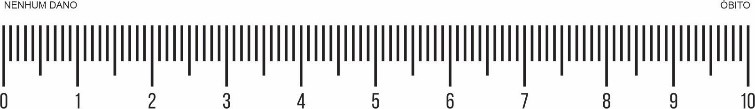 | | | |  |
| 38 | Metoclopramide (1 ampoule of 2mL - 5mg/mL) was prescribed. The Dilution manual guides you to administer direct IV for 1 to 2 min without dilution. It was done in 10 seconds. | | | | | 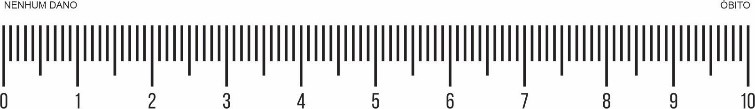 | | | |  |
| 39 | Metoclopramide (1 ampoule of 2m - 5mg/mL) was prescribed. The manual guides undiluted direct IV administration from 1 to 2 minutes. It was administered in one hour and 10 minutes. | | | | | 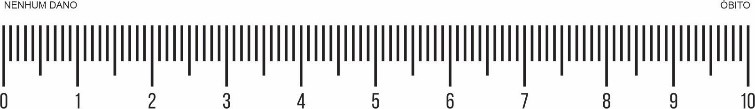 | | | |  |
| 40 | A 2 g of dipyrone was prescribed for intravenous administration. The dilution manual recommends direct IV administration very slowly and does not exceed 500 mg/min. It was done in 40 seconds. | | | | | 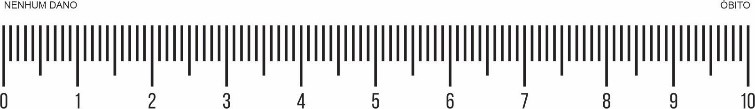 | | | |  |
| 41 | 1 g of dipyrone was prescribed for intravenous administration. The dilution manual recommends maximum infusion speed of 500 mg/min. It was done in three hours and 20 minutes. | | | | | 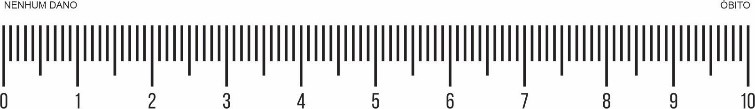 | | | |  |
| 42 | 1 g of dipyrone was prescribed for intravenous administration. The patient refused to use all the content because he was feeling pain in access. | | | | | 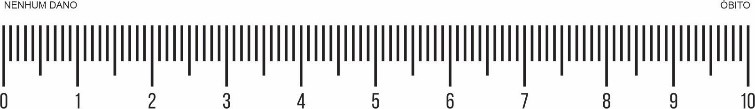 | | | |  |
| 43 | Cefazoline (1g) was prescribed for intravenous administration. The dilution manual guides reconstitution in 10mL of water for injection, with expansion to 10.6mL and direct intravenous administration between 3-5 minutes, according to the KEFAZOL package leaflet ®. Administration was made in about 32 seconds. | | | | | 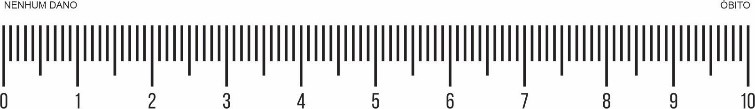 | | | |  |
| 44 | Cefazoline (1g) was prescribed for intravenous administration. The dilution manual recommends the time of direct intravenous administration between 3 - 5 minutes and does not recommend diluting in saline 0.9%. Done in 56 seconds and reconstitution in 10ml of SF 0.9%. | | | | | 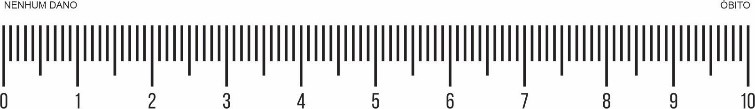 | | | |  |
| 45 | | | Patient using AAS (100 mg tablet). Patient had cerebral cardiovascular accident and technique preferred to dissolve and dilute the pill of the drug. Patient was swallowing food, liquids and pills normally. | | | | 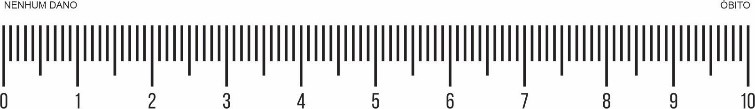 | | | |
| 46 | | | Vancomycin (1g) for intravenous administration was prescribed. Upon reaching the patient's bed, the technique verifies that the previous dose had been prepared in 250mL of 0.9% saline solution. It returned to the post and inspired 0.9% saline volume needed to reach the volume of 250mL,  because he had prepared the drug with 100 mL of SF 0.9%. | | | | 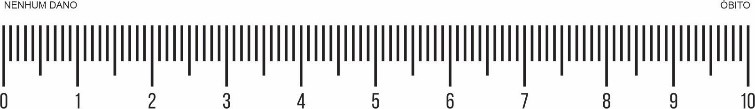 | | | |
| 47 | | | Vancomycin (1g) has been prescribed for intravenous administration. The dilution manual guides infuse in 2 hours. Done in an hour. | | | | 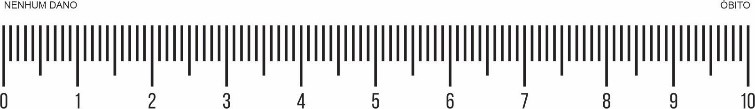 | | | |
| 48 | | | Omeprazole (40 mg) has been prescribed for intravenous administration. The dilution manual instructs you to infuse a maximum of 4ml/min. It was done between 15 and 18 seconds. | | | | 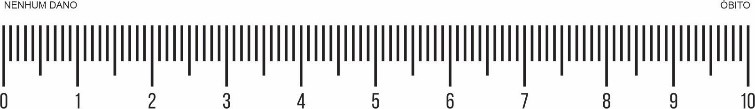 | | | |
| 49 | | | Ketoprofen (100 mg) has been prescribed for intravenous administration. The dilution manual guides reconstitution with water for injection and administration time of 20 minutes. It was reconstituted in 100 ml of SF 0.9% and administered in 51 minutes and 28 seconds (39 drops/minute). | | | | 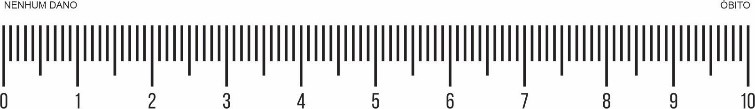 | | | |
| 50 | | | Bromopride (5mg) has been prescribed for intravenous administration. The dilution manual guides infusion slowly for more than 3 minutes. Done in 43 seconds. | | | | 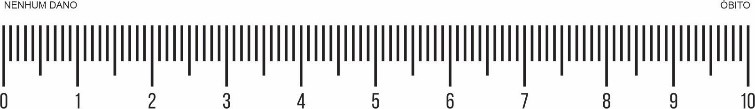 | | | |
| 51 | | | Clindamycin (600 mg) has been prescribed for intravenous administration. The dilution manual guides you to dilute 600mg in 50ml and infuse in 20min. It was diluted in 100ml and done in 40 minutes. | | | | 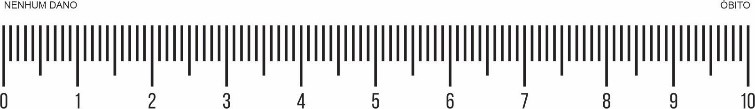 | | | |
| 52 | | | Hyoscin (20 mg) has been prescribed for intravenous administration. Recommended to administer 1mL/min, without dilution, as guided by the dilution manual. Administered in about 15 seconds (1 ml of the medicine + 9 ml of SF 0.9%). | | | | 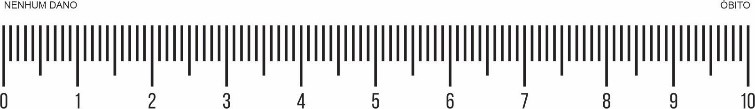 | | | |
| 53 | | | Cefepime (1g) has been prescribed for intravenous administration. The dilution manual instructs infuse 30 minutes. It was done in two hours. | | | | 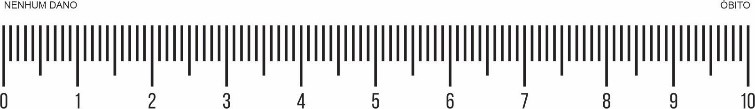 | | | |
| 54 | | | Ceftriaxone (1g) has been prescribed. The manual recommends IV infusion between 15-30 minutes, done in about 85 minutes (60 drops/minute). The medicine has been infused with clindamycin and the manual informs you that incompatible for Y-infusion. | | | | 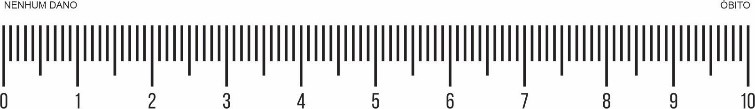 | | | |
| 55 | | | Ceftriaxone (1g) has been prescribed. Dilution manual directs IV direct between 2-4 minutes. Done in a minute. | | | | 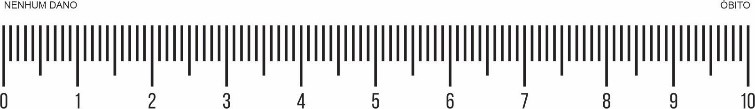 | | | |
| 56 | | | Oxacillin (2g) was prescribed for intravenous administration. A medicine usually diluted in 100 ml, 10 ml of water was aspirated for injection and used to reconstruct the four vials of ampoule using the same 10 ml syringe. Aspirated product reconstituted and diluted in SF 0.9% 250 ml. | | | | 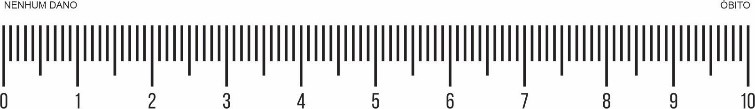 | | | |
| 57 | | Dexamethasone (16 mg) has been prescribed for intravenous administration. The dilution manual instructs you to infuse for 1 minute or more if necessary. was done in 30 seconds. | | | | | 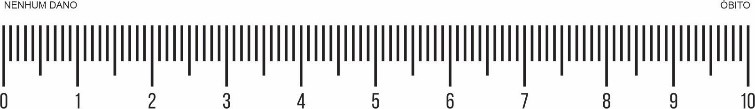 | | | |
| 58 | | Glucose (25%) was prescribed, 60 ml administration. The manual guides you that the 25% solution should be administered at 6 mL/minute. Made in 43 seconds syringe with 20mL. | | | | | 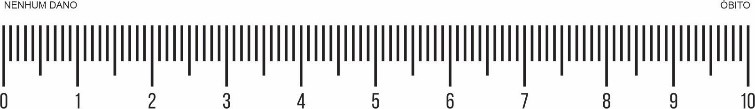 | | | |
| 59 | | Tramadol (100 mg) has been prescribed for intravenous administration. The dilution manual guides IV drop. About 10 drops/minute were done. | | | | | 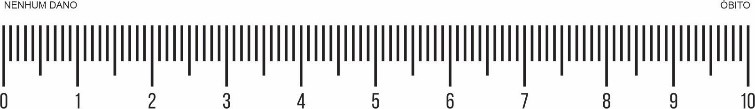 | | | |
| 60 | | Sulfasalazine (4 tablets of 500 mg) has been prescribed. The drug was prescribed for 8:00 a.m. and 4:00 p.m., and prescribed 12/12. It was administered at 6:00 p.m. | | | | | 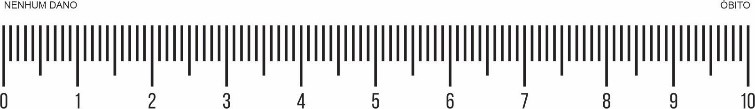 | | | |
| 61 | | Cefazoline (1g) was prescribed for intravenous administration. The administration was carried out one hour and four minutes after the time of the round. | | | | | 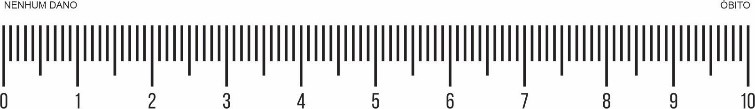 | | | |
| 62 | | Dipyrone 1 g was prescribed for injection at around 00:00 and was administered at 10:55 p.m.. | | | | | 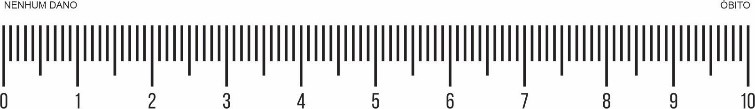 | | | |
| 63 | | Clonidine (1 tablet of 0.200 mg) was prescribed. The drug was taken to 8:00 p.m and was administered at 9:10 p.m, as prescription was sent to pharmacy at around 7:30 p.m. There were interruptions. Mistake wasn't from the infirmary. | | | | | 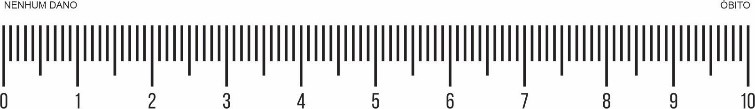 | | | |
| 64 | | Prescribed saline 0.9% 500 ml vial at 16:00 hours. It was made in the round at 17:32h. | | | | | 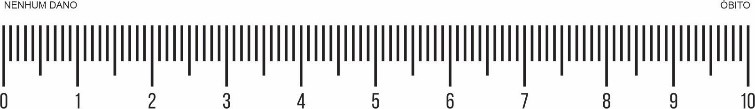 | | | |
| 65 | | Fluoxetine (20 mg) has been prescribed.  The patient preferred to take medication after the snack,  delaying it by more than an hour of the scheduled time. | | | | | 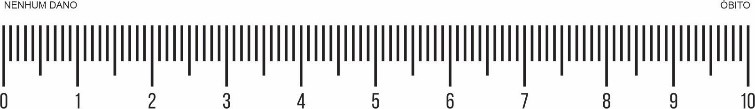 | | | |
| 66 | | Vancomycin 1g has been prescribed. Problems with patient access delayed the start of administration from 14:50 hours to 16:05 hours | | | | | 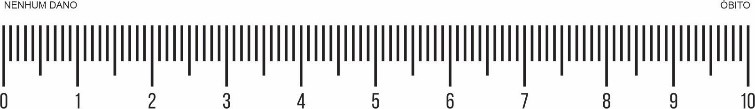 | | | |
| 67 | | Enalapril 10 mg prescribed and best-for-use at 10 a.m. It was administered at 11:40 a.m. | | | | | 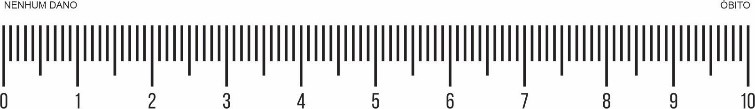 | | | |
