# Supplementary material for: Assessing the severity of medication administration errors identified in an observational study using a valid and reliable method
Source: J Pharm Policy Pract. 2023 Nov 14;16:143. doi: 10.1186/s40545-023-00653-x (PMC10648330; doi:10.1186/s40545-023-00653-x)
Supplement: Supplementary file 2 — Additional file 2. Guidance letter to judges. [file 40545_2023_653_MOESM2_ESM.docx]

**Additional file 2** - Guidance letter to judges

Name Date

Title E-mail

Dear: _______

Thank you for agreeing to collaborate in the process to assess the significance of administration errors. The scale was validated in Brazil to assess the severity of errors identified in this study of incidence of medication administration errors.

Brief descriptions of sixty seven errors that resulted in patients not receiving the prescribed drugs as planned are included here. Please classify each of them in terms of its potential clinical significance. The scale ranges from zero to ten, where zero should be given to an incident that would not result in effects on the patient and ten to one incident that would result in the death of the patient.

Mark the scale clearly by circling the appropriate number or placing a clear mark anywhere between the numbers, as shown below. Suppose all patients are adult hospitalized general or surgical wards. It is critical to record how long it will take you to complete the assessment of all fifty cases. If you have any additional comments, include them in the space provided.


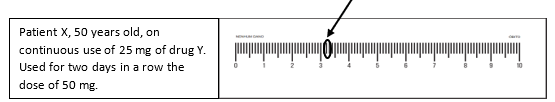


I requested the participation of different professionals with different backgrounds and academic degrees, so that a wide range of health professionals is represented. Your answers are important, so evaluate the cases individually. All responses will be anonymous and grouped with those of other health professionals to produce an average response for each case.

Please reply to the score form within two weeks and if you have any questions, please do not hesitate to contact me at (71) 991642210 or [by (author](mailto:lindembergrn@gmail.com) information).

Thank you very much for your collaboration.
