# Supplementary material for: Assessing the severity of medication administration errors identified in an observational study using a valid and reliable method
Source: J Pharm Policy Pract. 2023 Nov 14;16:143. doi: 10.1186/s40545-023-00653-x (PMC10648330; doi:10.1186/s40545-023-00653-x)
Supplement: Supplementary file 4 — Additional file 4. Categories of Medication Errors. [file 40545_2023_653_MOESM4_ESM.docx]

**Additional file 4 –** Categories of Medication Errors

- **Omission:** A dose of medication that has not been administered by the time of the next scheduled dose. Doses according to physician instructions, nurse clinical judgment, or the absence of the patient from the ward, are not included in this category.
- **Unprescribed dose:** The administration of a drug dose that was never prescribed for the patient. It is classified as a wrong drug if drug X was given instead of the prescribed drug Y.
- **Extra (dose):** The administration of an additional dose to the prescribed medicine. It includes taking the medicine more times a day than prescribed and taking another dose when the prescription is terminated.
- **Dose (wrong):** Any dose of a correct drug via the correct route, but in a different amount than prescribed (Inappropriate amount or number). For injectable drugs, any dose that is ±10% or more of the correct dose; for any other pharmaceutical form, any dose that is ±17% or more of the correct dose in the observer's judgment. In the judgment of doses, the measurements obtained with devices or appliances usually used in the institution should be considered (graduation in syringes, dosing burette, dropper, etc.)
- **Route (wrong):** The administration of a correct drug via a route or place of administration that differs from the prescription. Administration of a drug via the oral route when the prescription required the intramuscular route. Included in this category is the administration of eye drops to the left eye when it was prescribed to be applied to the right eye.
- **Pharmaceutical form (wrong):** The administration of the correct dose of a drug via the correct route, but not prescribed in a pharmaceutical form, especially when this has been specified. Included in this category is the administration of a slow-release pharmaceutical form when a rapid release form had been prescribed.
- **Technique (wrong):** Exclusion or inadequate performance of a prescribed procedure immediately before the administration of each dose. For example, taking a pulse before administering a beta-blocker.
- **Time** **(wrong):** the administration of a dose more than 60 min before or after the time scheduled by the nurse. For medications prescribed to be taken before, after, or at food, the administration of a dose more than 30 min before or after food. The time for comparison is the time used by the nurse in the prescription.
