# Supplementary material for: Assessing the severity of medication administration errors identified in an observational study using a valid and reliable method
Source: J Pharm Policy Pract. 2023 Nov 14;16:143. doi: 10.1186/s40545-023-00653-x (PMC10648330; doi:10.1186/s40545-023-00653-x)
Supplement: Supplementary file 5 — Additional file 5. Grouping MAE. [file 40545_2023_653_MOESM5_ESM.docx]

| **Error** | **Drug involved** | **Description** | **Average score** | **Severity** |
| --- | --- | --- | --- | --- |
| **100** | Fluoxetine (tablet 20 mg) | Patient preferred to take medication after snacking. | 2,6 | Minor |
| **47** | Dimeticone (tablet 40 mg) | Prescribed drops, administered tablet | 2,8 | Minor |
| **18** | calcium carbonate + vitamin D tablet (1250 mg + 400 IU) | Observador reports that he followed the round of the technique and this drug was not administered, but was checked. | 2,9 | Minor |
| **41** | Codeine 30mg + Paracetamol 500mg (tablet) | The patient was not in bed. I didn't watch the technique return at a later time, nor was I checked. | 2,9 | Minor |
| **99** | Spironolactone (tablet 100 mg) | Observer reports that he accompanied the technique at 9:30 a.m., when she medicated patients at 8 a.m. and 10 a.m. After that he went to give baths and no longer medicated until 12 noon, medicine of the round of 10 am. Técnica was responsible for six beds, and the census was 21/23. | 2,9 | Minor |
| **112** | Hyabak ® (ophthalmic solution 0.15%) | Administered a surplus drop in the right eye; prescribed one drop in each eye of 6/6h. | 2,9 | Minor |
| **113** | Hyabak ® (ophthalmic solution 0.15%) | Administered a surplus drop into the left eye; prescribed a drop in each eye for 6/6h. | 2,9 | Minor |
| **114** | Hyabak ® (ophthalmic solution 0.15%) | Administered a surplus drop into the left eye; prescribed a drop in each eye for 6/6h. | 2,9 | Minor |
| **176** | Saline solution 0.9% (500ml) | Prescrito 1500ml/24h = 500ml/8hs. Aprazado para 16h e feito na ronda das 18h às 17h32. | 2,9 | Minor |
| **59** | Dipyrone (for injection 500 mg/ml) | Patient refused to use all content because he was experiencing pain in access. | 2,9 | Minor |
| **200** | Dimeticone (tablet 40 mg) | Scheduled for 2 p.m. and administered at 3:40 p.m. | 2,9 | Minor |
| **201** | Dimeticone (tablet 40 mg) | Scheduled for 2 p.m. and administered at 3:15 p.m. | 2,9 | Minor |
| **202** | Tramadol (vial 100 mg/2 ml) | Scheduled for 4 p.m. and administered at 6:15 p.m. | 2,9 | Minor |
| **203** | Tramadol (vial 100 mg/2 ml) | Scheduled for 2 p.m. and administered at 6:35 p.m. | 2,9 | Mnor |
| **65** | Dipyrone (for injection 500 mg/ml) | Medication scheduled for 24 hours, anticipated after consensus of the nursing team. | 2,9 | Minor |
| **50** | Dipyrone (for injection 500 mg/ml) | It was to have been administered in the 24-hour round and was administered 10:55 p.m. | 2,9 | Minor |
| **46** | [Dimenhydrinate](https://www.whocc.no/atc_ddd_index/?code=R06AA11) + Vit. B (for injection 30 mg/ml) | Interruption due to access. Medication prescribed as if necessary, orally. It was administered by intravenous infusion. | 3,5 | Moderate |
| **124** | Ivermectin (tablet 6 mg) | Prescribed 18mg, administered 12mg. | 3,5 | Moderate |
| **154** | Ondansentron (solution for injection 2 mg/ml) | Dilution manual indicates infusion between 15-30 minutes. Done in an hour and 40 minutes. | 3,7 | Moderate |
| **156** | Ondansentron (solution for injection 2 mg/ml) | Dilution manual guides infuse between 15-30 minutes. Done in 50 minutes. | 3,7 | Moderate |
| **157** | Ondansentron (solution for injection 2 mg/ml) | Dilution manual guides infuse between 15-30 minutes. Done in 45 minutes. | 3,7 | Moderate |
| **158** | Ondansentron (solution for injection 2 mg/ml) | Dilution manual guides administration time of 15-30 min. Done in one hour and 10 minutes. | 3,7 | Moderate |
| **159** | Ondansentron (solution for injection 2 mg/ml) | Dilution manual guides dilute in 50 ml and infuse in 15-30. Done in 31.7 minutes and before tramadol. | 3,7 | Moderate |
| **160** | Ondansentron (solution for injection 2 mg/ml) | Dilution manual guides administration time of 15-30 min. Done in 32 seconds. | 3,7 | Moderate |
| **161** | Ondansentron (solution for injection 2 mg/ml) | Infusion made in about 33 minutes. Dilution manual guides intermittent infusion of 15 to 30 minutes. Prescription guides administration before tramadol, was administered concomitantly with this drug. | 3,7 | Moderate |
| **162** | Ondansentron (solution for injection 2 mg/ml) | Medication prescribed if necessary for nausea and vomiting or before tramadol. The scheduling was 8/8h (16h/ 24h/ 08h). It was administered in the round of 10 p.m. to 11:01 p.m. Manual dilution recommends infusing for 15-30 minutes, leaflet Vonau ® guides dilute 50-100 mL SF 0.9% or SG5% and infuse for no less than 15 minutes. Diluted in 100 ml of 0.9% SF, 22 drops/25 seconds, i.e. 37.8 minutes. | 3,7 | Moderate |
| **32** | Ketoprofen (for injection 1g) | Dilution manual guides reconstitution with water for injection and administration time of 20 minutes. Reconstituted in 100 ml 0.9% SF and administered in 51 minutes and 28 seconds (39 drops/minute). There were two interruptions, one after installing the drug, had to stop to wash the access and the second, the resident doctor interrupted to examine patient. | 3,8 | Moderate |
| **109** | [Hyoscine plus dipyrone](https://www.google.com/search?sxsrf=APwXEdeavjqg9sEZRIA7yxJdVyW6AiZGhA:1681311845048&q=hyoscine+dipyrone&spell=1&sa=X&ved=2ahUKEwjz-Z7azqT-AhXdqpUCHeHrAmAQkeECKAB6BAgIEAE) (for injection, 5 ml) | Manual de diluição orienta infundir 1 ml/minuto e foi feito em uma hora e 17 minutos. Além disso, foi prescrito 3 ml ao invés de 2 ml, como foi feito. | 3,8 | Moderate |
| **135** | Metoclopramide (solution for injection 5 mg/ml) | Manual guides direct undiluted IV, from 1 to 2 minutes. Diluted in one hour and 10 minutes. | 3,8 | Moderate |
| **137** | Metoclopramide (solution for injection 5 mg/ml) | Manual guides direct undiluted IV, from 1 to 2 minutes. Scheduled for 2 p.m. and administered at 3 p.m. | 3,8 | Moderate |
| **155** | Ondansentron (solution for injection 2 mg/ml) | Scheduled for 10 p.m., administered at 11 p.m. | 3,8 | Moderate |
| **57** | Dipyrone (for injection 500 mg/ml) | Dilution manual recommends maximum infusion rate of 500 mg/min. Done in three hours and 20 minutes. | 3,9 | Moderate |
| **58** | Dipyrone (for injection 500 mg/ml) | Dilution manual recommends maximum infusion rate of 500 mg/min. Done in an hour and five minutes. | 3,9 | Moderate |
| **57** | Dipyrone (for injection 500 mg/ml) | Dilution manual recommends maximum infusion rate of 500 mg/min. Done in three hours and 20 minutes. | 3,9 | Moderate |
| **51** | Dipyrone (for injection 500 mg/ml) | Dilution manual guides maximum infusion rate of 500mg/min. Done in an hour and 30 minutes. | 3,9 | Moderate |
| **78** | Dipyrone (for injection 500 mg/ml) | During technical preparation, 9% saline solution was aspirated and did not aspire to the volume of dipyrone ampoules. By discarding waste, he noticed error and redid process. | 3,9 | Moderate |
| **11** | Bisacodyl (tablet 5 mg) | Medication from the round of 16h and administered at 16h08. At 4:36 p.m., an intervention was performed because the prescription was four tablets for preparation. | 4 | Moderate |
| **48** | Dimeticone (drops 75 mg/ml) | Prescribed 40 drops, made 45 drops | 4 | Moderate |
| **60** | Dipyrone (for injection 500 mg/ml) | Medication was not administered in the 12-hour round. The prescription was marked with observation if low blood pressure. (No pressure measurement obtained by the observer). | 4 | Moderate |
| **61** | Dipyrone (for injection 500 mg/ml) | Medication was not prepared by the technique, because during the administration of codeine tablet + paracetamol patient reported not feeling pain and technique did not administer dipyrone. Prescription rolling. | 4 | Moderate |
| **55** | Dipyrone (for injection 500 mg/ml) | It was prepared at 10:30 p.m. and discarded about 11:55 p.m. | 4 | Moderate |
| **5** | Amitriptyline (tablets 25 mg) | Administered with water. Drug scheduled for 20h and administered in the round of 22h, prescribed dose of 50 mg (02 tablets). Patient reported that dose was wrong, technique was to check prescription and did not return. | 4 | Moderate |
| **45** | [Dimenhydrinate](https://www.whocc.no/atc_ddd_index/?code=R06AA11) + Vit. B (for injection 30 mg/ml) | Prescribed to administer 50mg of Dimenhydrinate, but was made 30mg (1 ampoule). The prescription was oral and was modified from pen change to intravenous (prescription error). | 4,2 | Moderate |
| **1** | Acetylsalicylic Acid (tablet 100 mg) | The patient had a stroke and the technique preferred to dilute medication. Patient was swallowing food, liquids and tablets. | 4,3 | Moderate |
| **13** | Bromopride (vial 5 mg/ml) | Patient complaining of pain in the access. Administration was interrupted. The prescription was designed with observation of no access. | 4,3 | Moderate |
| **183** | Tramadol (solution for injection, 100 mg/2 ml) | Medication prescribed as if necessary. Manual recommends IV infusion drop by drop, made about 30 drops / minute, that is, more than 60 minutes. As there was disregard of the volume of solution of the Ji system that contained the drug, despite the small volume, the impact of the loss cannot be specified. Error in the preparation process. | 4,5 | Moderate |
| **34** | Clindamycin (vial 600 mg) | Manual dilution guides dilute 600mg in 50ml and infuse in 20min. Diluted in 100ml and made in 40 minutes. | 4,5 | Moderate |
| **35** | Clindamycin (vial 600 mg) | Manual dilution guides dilute 600mg in 50ml and infuse in 20min. Diluted in 100ml and made in 30 minutes. | 4,5 | Moderate |
| **33** | Clindamycin (vial 600 mg) | Dilution manual recommends administration time of 600mg intravenous 20 minutes and dilution in 50mL of 0.9% saline, glucose serum 5%. It was diluted in 100 ml in a time of 27.39 minutes (73 drops/min). | 4,5 | Moderate |
| **12** | Bromopride (vial 5 mg/ml) | Dilution manual guides direct IV in 3 minutes. Done in one hour and 10 minutes. | 4,6 | Moderate |
| **14** | Bromopride (vial 5 mg/ml) | Manual dilution guides infusion time slowly exceeding 3 minutes. Done in 43 seconds. | 4,6 | Moderate |
| **15** | Bromopride (vial 5 mg/ml) | Dilution manual guides time of administration of intermittent intravenous infusion slowly (greater than 3 min). Done in 38 seconds. | 4,6 | Moderate |
| **16** | Bromopride (vial 5 mg/ml) | Patient in allergic crisis and agitated. Dilution manual recommends IV intermittent infusion slowly (more than 3 minutes). Same recommendation given in the package leaflet of the reference medicine, Digesan ® . Done in about 30 seconds. | 4,6 | Moderate |
| **90** | Enalapril (tablet 10 mg) | Administration for round 10h, done 11h40. Technician responsible for four beds, being census 12/21. | 4,6 | Moderate |
| **179** | Saline solution 0.9% (500ml) + glucose 25% (solution for injection) | Aspirated four ampoules of glucose 25% and added to the serum 500 ml. Serum volume was not adjusted with the addition of the 40 ml volume. Drug time of 20drops/minute. | 4,8 | Moderate |
| **115** | Ibuprofen (oral suspension drops, 50 mg/ml) | Technique did not position dropper bottle in the vertical position and there was no gout formation. From what was dosed, administered dose was incorrect. | 4,8 | Moderate |
| **116** | Ibuprofen (oral suspension drops, 50 mg/ml) | The accounting of the dose was impaired because the technique did not position the dropper correctly and there was no formation of drops. | 4,8 | Moderate |
| **115** | Ibuprofen (oral suspension drops, 50 mg/ml) | Technique did not position dropper bottle in the vertical position and there was no gout formation. From what was dosed, administered dose was incorrect. | 4,8 | Moderate |
| **116** | Ibuprofen (oral suspension drops, 50 mg/ml) | The accounting of the dose was impaired because the technique did not position the dropper correctly and there was no formation of drops. | 4,8 | Moderate |
| **166** | Oxacillin (for injection 500 mg) | Problems with patient access delayed administration by an hour, in the round of 16h, was administered 17h10. Medication time scheduled for 25 minutes. | 4,8 | Moderate |
| **129** | Meropenem (Vial 500 mg) | In the round of 08h it was necessary to change access of the patient, so medication was administered by another technique at 09h22 that made this exchange. Dilution manual recommends IV infusion administration time between 15-30 minutes, done in 32 minutes and 26 seconds (62 drops/minute). | 4,8 | Moderate |
| **131** | Meropenem (Vial 500 mg) | Scheduled for 4 p.m., administered on the 6 p.m. round, at 5:45 p.m. Technique present since the round of 08h and responsible for four beds, being census 22/23. | 4,8 | Moderate |
| **22** | Cefazoline (for injection 1g) | Administration was carried out one hour and four minutes after the time of the round. Observer informs in observations that dilution manual recommends direct intravenous administration time between 3 and 5 minutes, but does not inform how long the professional administered the drug. | 4,8 | Moderate |
| **26** | Cefazoline (for injection 1g) | It was to have been administered in the 24-hour round and was administered 10:55 p.m. | 4,8 | Moderate |
| **174** | Saline solution 0.9% (500ml) | Prescrito 1500ml/24h = 500ml/8hs. Feito em aproximadamente quatro horas. | 5 | Moderate |
| **175** | Saline solution 0.9% (500ml) | Prescrito 21 gotas/minuto. Feito 42 gotas/minuto. | 5 | Moderate |
| **108** | [Hyoscine plus dipyrone](https://www.google.com/search?sxsrf=APwXEdeavjqg9sEZRIA7yxJdVyW6AiZGhA:1681311845048&q=hyoscine+dipyrone&spell=1&sa=X&ved=2ahUKEwjz-Z7azqT-AhXdqpUCHeHrAmAQkeECKAB6BAgIEAE) (for injection, 5 ml) | Manual de diluição orienta infundir 1 ml/minuto e foi feito em uma hora e 17 minutos. Além disso, foi prescrito 3 ml ao invés de 2 ml, como foi feito. | 5 | Moderate |
| **167** | Potassium permanganate solution (1:80.000) | Technique records that it does not have, but on duty the day before I requested medication for the patient and confirmed the receipt with the same later. | 5 | Moderate |
| **181** | Sulfasalazine (tablet 500 mg) | Medication scheduled for 08h and 16h, prescribed from 12/12. Administered 6 p.m. | 5,1 | Moderate |
| **7** | Amitriptyline (tablet 25 mg) | Administered with water. Medication scheduled for 20h, administered 22h07 in the round of 22h. | 5,1 | Moderate |
| **132** | Mesalazine (tablet 400 mg) | It was not administered in the round of 08h, because there was only one drug in the patient's box. Full dose was sent by the pharmacy around 10 a.m. and administered 10:35 a.m. | 5,1 | Moderate |
| **187** | Vancomycin (for injection, 500 mg) | Dilution manual guides infuse in 2 hours. Done in an hour. | 5,1 | Moderate |
| **188** | Vancomycin (for injection, 500 mg) | Dilution manual guides infuse in 2 hours. Risk of red neck man syndrome. Done in about 41 minutes, 120 drops/minute. | 5,1 | Moderate |
| **189** | Vancomycin (for injection, 500 mg) | Dilution manual guides infuse in 2 hours. Done in an hour and a half. | 5,1 | Moderate |
| **191** | Vancomycin (for injection, 500 mg) | Dilution manual guides infuse in 2h. Done in an hour and a half. | 5,1 | Moderate |
| **94** | Enoxaparin (preloaded syringe 40 mg) | Administered at 2 p.m. to 3:50 p.m. Técnica was responsible for four beds, and the census was 19/21. | 5,2 | Moderate |
| **95** | Enoxaparin (preloaded syringe 40 mg) | Scheduled for round of 08h, administered 09h27. Técnica was responsible for six beds, and the census was 21/23. | 5,2 | Moderate |
| **96** | Enoxaparin (preloaded syringe 40 mg) | Administered on the 4 p.m. round, at 5:05 p.m. Técnica was responsible for three beds, the census being 17/23. | 5,2 | Moderate |
| **97** | Enoxaparin (preloaded syringe 40 mg) | Medication was checked and was scheduled for 10 a.m. Administered in the round of 08h, to 08h15. | 5,2 | Moderate |
| **98** | Enoxaparin (preloaded syringe 40 mg) | Medication was checked and was scheduled for 10 a.m. Administered in the round of 08h, to 08h15. | 5,2 | **Moderate** |
| **177** | Saline solution 0.9% (500ml) | Medicamento checado as 02h, porém não foi instalado nesse horário, nem em parte da ronda de 04h, uma vez que a dose anterior não estava com a vazão correta e não havia terminado ainda. | 5,2 | Moderate |
| **2** | Acetylsalicylic Acid (tablet 100 mg) | Medication scheduled for 2 p.m. and administered at 4:34 p.m. | 5,2 | Moderate |
| **3** | Folic Acid (tablet 5 mg) | Medication scheduled for 20h and was administered 21h10, because prescription went to pharmacy in the round of 19h30. There were interruptions. Mistake was not from the infirmary. | 5,2 | Moderate |
| **8** | Amlodipine (tablet 5 mg) | Medication of the round of 08h and was administered 09h15. | 5,2 | Moderate |
| **38** | Clonidine (tablet 0,200 mg) | Medication scheduled for 20h and was administered 21h10, because prescription went to pharmacy in the round of 19h30. There were interruptions. Mistake was not from the infirmary. | 5,2 | Moderate |
| **43** | Vitamin B-Complex (tablet) | Medication scheduled for 20h and was administered 21h10, because prescription went to pharmacy in the round of 19h30. There were interruptions. Mistake was not from the infirmary. | 5,2 | Moderate |
| **104** | Hydralazine (tablet 150 mg) | Medication scheduled for 20h and was administered 21h10, because prescription went to pharmacy in the round of 19h30. There were interruptions. Mistake was not from the infirmary. | 5,2 | Moderate |
| **125** | Lactulose (syrup 667mg/ml) | Scheduled for 2 p.m., administered at 3:50 p.m. | 5,2 | Moderate |
| **169** | Ranitidine (tablet 25 mg) | Medication scheduled for 20h and was administered 21h40, because prescription went to pharmacy in the round of 19h30. There were interruptions. Mistake was not from the infirmary. | 5,2 | Moderate |
| **170** | Lactate Ringer's KCl; NaCl; Glucose; MgSO4 (solution for injection) | Prescribed 7 drops/minute, i.e. 21 mL/h (23.8 hours). Done 6 drops/minutes, i.e. 27.8 hours. | 5,2 | Moderate |
| **184** | Tramadol (solution for injection, 100 mg/2 ml) | Very slow drip, then very slow infusion of a drug that needs earlier effect. | 5,2 | Moderate |
| **185** | Tramadol (solution for injection, 100 mg/2 ml) | Dilution manual guides IV drop. Made about 10 drops/minute. | 5,2 | Moderate |
| **19** | Cefazoline (for injection 1g) | Dilution manual recommends direct intravenous administration time between 3-5 minutes. Done in 35 seconds. | 5,2 | Moderate |
| **20** | Cefazoline (for injection 1g) | Dilution manual recommends direct intravenous administration time between 3-5 minutes. Done in 35 seconds. | 5,2 | Moderado |
| **23** | Cefazoline (for injection 1g) | Dilution manual guides reconstitution in 10mL of water for injection, with expansion to 10.6mL and direct intravenous administration between 3-5 minutes, corroborating the KEEFAZOL® package insert. Administration was done in about 32 seconds. | 5,2 | Moderate |
| **19** | Cefazoline (for injection 1g) | Dilution manual recommends direct intravenous administration time between 3-5 minutes. Done in 35 seconds. | 5,2 | Moderate |
| **130** | Meropenem (Vial 500 mg) | Manual dilution guides infuse between 15-30 minutes, done in an hour. There were interruptions. | 5,2 | Moderate |
| **193** | Dapsone (tablet 100 mg) | Patient preferred to take medication after snacking. Scheduled for 8 a.m. and administered at 10:26 a.m. | 5,3 | Moderate |
| **6** | Amitriptyline (tablet 25 mg) | Not administered in the 20h round, which the drug was agreed. I followed round and saw no dose of the drug. Medication found in the patient's box. | 5,3 | Moderate |
| **148** | Metoclopramide (solution for injection 5 mg/ml) | Prescribed tablet orally and administered intravenously for 23 seconds. | 5,6 | Moderate |
| **149** | Metoclopramide (solution for injection 5 mg/ml) | Prescribed tablet orally and administered intravenously for 23 seconds. | 5,6 | Moderate |
| **150** | Metoclopramide (solution for injection 5 mg/ml) | Prescribed tablet orally and administered intravenously for 23 seconds. | 5,6 | Moderate |
| **136** | Metoclopramide (solution for injection 5 mg/ml) | Manual guides direct undiluted IV, from 1 to 2 minutes. Diluted in 35 seconds. | 5,7 | Moderate |
| **92** | Enoxaparin (preloaded syringe 40 mg) | Administered quickly. Prescribed 70 mg, technique should adjust the dose with the syringe of 60 mg graduated EU (line 108-is this expression correct?). An incorrect technique was used in the adjustment, and a dose of 50 mg in the syringe was not guaranteed. Observer did not intervene. | 5,5 | Moderate |
| **93** | Enoxaparin (preloaded syringe 40 mg) | Manual dilution guides IV administration time – very slowly (do not exceed 500mg/min). Administered quickly. | 5,5 | Moderate |
| **91** | Enoxaparin (preloaded syringe 40 mg) | Administered quickly. Prescribed 70 mg, technique should adjust the dose with the 60 mg EU graduated syringe (line 108-is this expression correct?). Incorrect technique was used in the adjustment, not guaranteed dose of 50 mg in the syringe. Observer did not intervene. | 5,5 | Moderate |
| **27** | Cefepime (for injection 1g) | Manual dilution guides infuse 30 minutes. Done in two hours. | 5,5 | Moderate |
| **28** | Cefepime (for injection 1g) | Manual dilution guides infuse in 30 minutes. Drip infusion exceeded this time. | 5,5 | Moderate |
| **151** | Omeprazole (for injection, 40 mg) | Dilution manual guides to infuse a maximum of 4ml/min. Done in 15 seconds. | 5,6 | Moderate |
| **152** | Omeprazole (for injection, 40 mg) | Dilution manual guides to infuse a maximum of 4ml/min. Done in 40 seconds. | 5,6 | Moderate |
| **153** | Omeprazole (for injection, 40 mg) | It was prescribed by the physician on duty due to the patient's intercurrence. Professional did not follow dilution manual for preparation. | 5,6 | Moderate |
| **110** | Hyoscine (for injection 20 mg/ml, 1 ml) | Prescrito 10mg via oral para o dia dessa administração, 06.02.19. No dia anterior, 05.02, estava prescrito EV. Foi feito pela via endovenosa no tempo de 35 segundos. | 5,6 | Moderate |
| **111** | Hyoscine (for injection 20 mg/ml, 1 ml) | Interrupção do enfermeiro passando queixas da paciente que foi admitida no dia na unidade. Ao invés de administrar 1mL/min, sem diluição como orienta o manual de diluição, fez em cerca de 15 segundos as 1 ml da solução + 9 ml de SF 0,9%. | 5,6 | Moderate |
| **192** | Omeprazole (for injection, 40 mg) | Dilution manual: infuse maximum 4ml/min. Done in 18 seconds. | 5,6 | Moderate |
| **101** | Furosemide (for injection 10 mg/ml) | Manual dilution guides infuse between 1 and 2 min, done between 10-11 seconds. | 5,6 | Moderate |
| **102** | Furosemide (for injection 10 mg/ml) | Manual dilution advises infusing between 1 and 2 min, made between 18 seconds. | 5,6 | Moderate |
| **4** | Albumin (20%) 50 ml vials | 1st and 2nd vials in 30 min (15 min each) 3rd bottle in 35 min / 4th to 7th vial in 1h45 (approximately 25min each). Manual dilution guides from 1 to 2 ml/min = Minimum of 25 minutes each. Brushed equipment aspirating, due to the difficulty of fluidity, what remained in the syringe was administered direct IV, approximately 5 ml. There were interruptions. | 5,6 | Moderate |
| **139** | Metoclopramide (solution for injection 5 mg/ml) | Dilution manual recommends undiluted direct IV for 1 to 2 minutes; IV intermittent infusion in 15 minutes (50 ml of compatible solution). Administration done in 45 seconds. | 5,7 | Moderate |
| **140** | Metoclopramide (solution for injection 5 mg/ml) | Dilution manual guides direct IV administration time without dilution, for 1-2 minutes. Done in 30 seconds. | 5,7 | Moderate |
| **141** | Metoclopramide (solution for injection 5 mg/ml) | Dilution manual guides time of direct IV administration without dilution, for 1-2 minutes. Done in about 40 seconds. | 5,7 | Moderate |
| **142** | Metoclopramide (soluction for injection 5 mg/ml) | Dilution manual guides direct IV administration time without dilution, for 1-2 minutes. Done in about 50 seconds. | 5,7 | Moderate |
| **143** | Metoclopramide (solution for injection 5 mg/ml) | Dilution manual guides direct IV administration time without dilution, for 1-2 minutes. Done in 30 seconds. | 5,7 | Moderate |
| **144** | Metoclopramide (solution for injection 5 mg/ml) | Dilution manual guides time of direct IV administration without dilution, for 1-2 minutes. Done in about 40 seconds. | 5,7 | Moderate |
| **145** | Metoclopramide (solution for injection 5 mg/ml) | Manual guides direct IV undiluted, for 1 to 2 minutes and prescribed slowly. Administered in 42 seconds with leftover small volume in a syringe. | 5,7 | Moderate |
| **146** | Metoclopramide (solution for injection 5 mg/ml) | Dilution manual guides direct IV administration time without dilution, for 1-2 minutes. Done in 45 seconds time. | 5,7 | Moderate |
| **139** | Metoclopramide (solution for injection 5 mg/ml) | Dilution manual recommends undiluted direct IV for 1 to 2 minutes; IV intermittent infusion in 15 minutes (50 ml of compatible solution). Administration done in 45 seconds. | 5,7 | Moderate |
| **133** | Metoclopramide (solution for injection 5 mg/ml) | Dilution Manual guides direct IR for 1 to 2 min without diluting. It was done in 10 seconds. | 5,7 | Moderate |
| **134** | Metoclopramide (solution for injection 5 mg/ml) | Manual guides direct undiluted IV, from 1 to 2 minutes. Diluted in 16 seconds. | 5,7 | Moderate |
| **133** | Metoclopramide (solution for injection 5 mg/ml) | Dilution Manual guides direct IR for 1 to 2 min without diluting. It was done in 10 seconds. | 5,7 | Moderate |
| **140** | Metoclopramide (solution for injection 5 mg/ml) | Dilution manual guides direct IV administration time without dilution, for 1-2 minutes. Done in 30 seconds. | 5,7 | Moderate |
| **163** | Oxacillin (for injection 500 mg) | Dilution manual recommends IV administration time infusion between 15 - 30 minutes, was done in 35.7 minutes (56 drops/minute). | 5,7 | Moderate |
| **164** | Oxacillin (for injection 500 mg) | Connected bottle in equipment that was used in previous dose in bed. Dilution manual recommends IV administration time infusion between 15 - 30 minutes. Done in 41.66 minutes (48 drops/minute). | 5,7 | Moderate |
| **165** | Oxacillin (for injection 500 mg) | A drug usually diluted in 100 ml, 10 ml of water was aspirated for injection and used to reconstitute the four vials using the same 10 ml syringe. Aspirated product reconstituted and diluted in SF 0.9% 250 ml. Lack of SF 0.9% in the unit due to insufficient order. Técnica refuses to remove a volume of 0.9% 250 ml SF and verbalizes this information. There is no time error, the round is 22h and the drug was administered 21h49. It would be a technical error because it did not follow the dilution manual. | 5,7 | Moderate |
| **126** | Losartan (tablet 50 mg) | Patient reports that he uses losartan because when he is in pain his pressure rises. As the blood pressure was at 100x70 mmHg and without pain at the time, the technique chooses not to administer the drug. | 5,8 | Moderate |
| **127** | Losartan (tablet 50 mg) | BP = 96x46mmHg (only considerable information explaining the error is pressure, there are no observations made by the observer). Bruna's thought: due to the patient's pressure being low, probably technician did not want to administer the dose. However, it should be analyzed if the pressure is controlled or really low, because being the latter, it is necessary to adjust the dose or change medication for a weaker one. | 5,8 | Moderate |
| **126** | Losartan (tablet 50 mg) | Patient reports that he uses losartan because when he is in pain his pressure rises. As the blood pressure was at 100x70 mmHg and without pain at the time, the technique chooses not to administer the drug. | 5,8 | Moderate |
| **127** | Losartan (tablet 50 mg) | BP = 96x46mmHg (only considerable information explaining the error is pressure, there are no observations made by the observer). Bruna's thought: due to the patient's pressure being low, probably technician did not want to administer the dose. However, it should be analyzed if the pressure is controlled or really low, because being the latter, it is necessary to adjust the dose or change medication for a weaker one. | 5,8 | Moderate |
| **17** | Captopril (tablet 25 mg) | It was not administered due to low BP (8x6). I had no observation in the prescription not to do. | 5,8 | Moderate |
| **128** | Losartan (tablet 50 mg) | Assitent did not administer the Losartan tablet because he judged that the patient's blood pressure was low and he was going to be referred to the Surgical Center in the next few hours. | 5,8 | Moderate |
| **168** | Propranolol (tablet 10 mg) | Medication was not administered at around 8 a.m. due to blood pressure. He waited to check it again, but medicine was not administered. The prescription was marked with observation if low blood pressure. (BP= 100x60 mmHg). | 5,8 | Moderate |
| **44** | Dexamethasone (for injection 4 mg/ml) | Manual dilution guides infuse for 1 minute or more, if necessary, done in 30 seconds. | 6 | Moderate |
| **190** | Vancomycin (for injection, 500 mg) | Upon reaching the patient's bed, the technique verifies that the previous dose had been prepared in 250mL of 0.9% saline. She returned to the clinic and aspired to the 0.9% volume of saline needed to reach the volume of 250mL, because she had prepared the drug with 100 mL of 0.9% saline. | 6 | Moderate |
| **21** | Cefazoline (for injection 1g) | Administration was performed one hour and four minutes after the time of the round. Observer informs in observations that dilution manual recommends time of direct intravenous administration between 3 and 5 minutes, but does not inform how long the professional administered the drug. | 6 | Moderate |
| **62** | Dipyrone (for injection 500 mg/ml) | Medication administered to another patient, but checked in the prescription of this patient | 6 | Moderate |
| **31** | Ceftriaxone (for injection 1g) | Dilution manual guides 1 g in 10 ml, C + 10 to 40 mg/ml. Direct IR between 2-4 minutes, done in one minute. There were interruptions. | 6,1 | Moderate |
| **9** | Atenolol (tablet 50mg) | Prescribed atenolol 50mg. Use 25mg orally. There were interruptions. | 6,1 | Moderate |
| **39** | Codeine 30mg + Paracetamol 500mg (tablet) | Prescribed codeine 30mg without association. Codeine 30mg+paracetamol 500mg is administered. | 6,3 | Moderate |
| **194** | Regular insulin (solution for injection) | Medication was not administered. Confirmed with patient that the same was not done. HGT = 233. | 6,3 | Moderate |
| **195** | Regular insulin (solution for injection) | Medication was not administered. Confirmed with patient that the same was not done. HGT = 249. | 6,3 | Moderate |
| **196** | Regular insulin (solution for injection) | The technician took NPH insulin aspirated before making HGT, after capillary glycemia, came back and aspired to regular insulin. Regular insulin SN (04 units). Technician checked the item of systematic use came up with the time of 08h and checked 22h. | 6,3 | Moderate |
| **197** | Regular insulin (solution for injection) | Drug not administered. | 6,3 | Moderate |
| **119** | Regular insulin (solution for injection, 100 UI/ml) | Prescribed 4ui of Insulin R for HGT = 190-250, HGT = 191 of the patient. It was not administered. | 6,3 | Moderate |
| **120** | Regular insulin (solution for injection, 100 UI/ml) | Prescribed 4 ui if HGT between 190 and 250. HGT = 209. Observer reports: I did not observe the technique do and was not checked. | 6,3 | Moderate |
| **121** | Regular insulin (soluction for injection, 100 UI/ml) | Regular insulin prescription, if necessary, as per HGT. HGT (10pm) | 6,3 | Moderate |
| **122** | Regular insulin (solution for injection, 100 UI/ml) | Medication was not administered. Confirmed with patient that the same was not done. HGT = 126. | 6,3 | Moderate |
| **123** | Regular insulin (solution for injection, 100 UI/ml) | Regular insulin is prescribed if HGT>190. Not checked | 6,3 | Moderate |
| **52** | Dipyrone (for injection 500 mg/ml) | Dilution manual guides maximum infusion rate of 500mg/min. Done in 46 seconds. | 6,3 | Moderate |
| **53** | Dipyrone (for injection 500 mg/ml) | Dilution manual guides maximum infusion rate of 500mg/min. Done between 27 - 35 seconds. | 6,3 | Moderate |
| **54** | Dipyrone (for injection 500 mg/ml) | Manual dilution guides maximum infusion rate of 500mg/min. Done in 35 seconds. | 6,3 | Moderate |
| **56** | Dipyrone (for injection 500 mg/ml) | Dilution manual recommends maximum infusion rate of 500 mg/min. Done between 20 and 50 seconds. | 6,3 | Moderate |
| **49** | Dipyrone (for injection 500 mg/ml) | Manual dilution recommends direct IV very slowly and do not exceed 500 mg/min. Done in 17 seconds. | 6,3 | Moderate |
| **63** | Dipyrone (for injection 500 mg/ml) | Manual recommends: IV – very slowly (do not exceed 500mg/min). Done in about 27 seconds. | 6,3 | Moderate |
| **64** | Dipyrone (for injection 500 mg/ml) | Dilution manual recommends IV IV administration time – very slowly (do not exceed 500mg/min). Done in 27 seconds. | 6,3 | Moderate |
| **66** | Dipyrone (for injection 500 mg/ml) | Dilution manual recommends IV IV administration time – very slowly (do not exceed 500mg/min). Done in 31 seconds. | 6,3 | Moderate |
| **67** | Dipyrone (for injection 500 mg/ml) | Medication prescribed if pain or fever; 1g intravenous up to 6/6h. The institution's dilution manual, as well as the Novalgina (R) package leaflet, advises to administer very slowly, not exceeding 500 mg/min. Done in 41 seconds. | 6,3 | Moderate |
| **69** | Dipyrone (for injection 500 mg/ml) | Manual dilution recommends direct IV very slowly and do not exceed 500 mg/min. Done in 64 seconds. | 6,3 | Moderate |
| **70** | Dipyrone (for injection 500 mg/ml) | Technique didn't wash her hands, she just put on her gloves. Technique tells me that it does not administer the entire volume of syringes to prevent air bubble from being administered. Administered between 30 and 50 seconds. | 6,3 | Moderate |
| **74** | Dipyrone (for injection 500 mg/ml) | Technique didn't wash her hands, she just put on her gloves. Technique tells me that it does not administer the entire volume of syringes to prevent air bubble from being administered. Administered in about 50 seconds. | 6,3 | Moderate |
| **75** | Dipyrone (for injection 500 mg/ml) | Technique didn't wash her hands, just put on her gloves. Técnica tells me that she does not administer the entire volume of syringes to prevent air bubbles from being administered. Administered in about 30 seconds. | 6,3 | Moderate |
| **76** | Dipyrone (for injection 500 mg/ml) | Manual recommends: IV very slowly (do not exceed 500mg/min); in the adjustment of syringe volume there was a disregard of unquantified amount of medication. Administration was in about 38 seconds. | 6,3 | Moderate |
| **77** | Dipyrone (for injection 500 mg/ml) | Manual recommends: IV very slowly (do not exceed 500mg/min); In the adjustment of syringe volume, there was disregard for the unquantified amount of medication. Administration was in about 32 seconds. | 6,3 | Moderate |
| **79** | Dipyrone (for injection 500 mg/ml) | Dilution manual recommends: IV – very slowly (do not exceed 500mg/min); In the adjustment of syringe volume, there was a disregard of the unquantified amount of medication. Done in about 40 seconds | 6,3 | Moderate |
| **80** | Dipyrone (for injection 500 mg/ml) | Manual dilution guides not to exceed rate of 500mg/min. Done in 20 seconds. | 6,3 | Moderate |
| **81** | Dipyrone (for injection 500 mg/ml) | Manual dilution guides IV administration time – very slowly (do not exceed 500mg/min). Done in 20 seconds. | 6,3 | Moderate |
| **82** | Dipyrone (for injection 500 mg/ml) | Medication scheduled, patient complaining of severe headache, despite the use of morphine 10 mg VO SN; Administration should not exceed 500 mg/min. Done in 75 seconds. | 6,3 | Moderate |
| **83** | Dipyrone (for injection 500 mg/ml) | Preparation of the drug 05h15. Manual dilution guides not to exceed rate of 500mg/min. Done in 40 seconds. | 6,3 | Moderate |
| **84** | Dipyrone (for injection 500 mg/ml) | 10 mL in 28 seconds. In 18 seconds, the remaining volume was administered10 mL in 28 seconds. In 18 seconds, the remaining volume was administered | 6,3 | Moderate |
| **85** | Dipyrone (for injection 500 mg/ml) | Prescribed Dipyrone 1g EV 6/6h (aprazado at 18/24/06/12); Check dose of 24h. The patient had lost access around 8 p.m., so part of the medication was administered at 8:28 p.m. (10 mL) and after changing access, the remaining volume was administered at 9:16 p.m. | 6,3 | Moderate |
| **86** | Dipyrone (for injection 500 mg/ml) | Technique didn't wash her hands, just put on her gloves. Técnica tells me that she does not administer the entire volume of syringes to prevent air bubbles from being administered. Administered in about 50 seconds. | 6,3 | Moderate |
| **87** | Dipyrone (for injection 500 mg/ml) | At the time the drug was administered, prescription was verbal. Medication is on prescription. Patient was just admitted. Injection was rapid, manual dilution orients not to exceed 500 mg/min. Done in 17 seconds. | 6,3 | Moderate |
| **89** | Dipyrone (for injection 500 mg/ml) | Dilution manual: Maximum infusion rate: 500mg/min. Done in about 40 seconds | 6,3 | Moderate |
| **39** | Codeine 30mg + Paracetamol 500mg (tablet) | Prescribed codeine 30mg without association. Codeine 30mg+paracetamol 500mg is administered. | 6,3 | Moderate |
| **36** | Clindamycin (for injection 150 mg/ml - 4 ml) | Technique didn't wash her hands, she just put on her gloves. Discards some volume of the drug from the system to remove air bubble from the equipment. Bedside. Used previously used equipment. | 6,6 | Moderate |
| **37** | Clindamycin (for injection 150 mg/ml - 4 ml) | Technician despises some volume for filling the system and removing air bubbles. As there was disregard of the volume of solution of the system that Ji contained medicine, despite small volume, it is not possible to specify the impact of the loss. Error in the preparation process. | 6,6 | Moderate |
| **103** | Glicose (solution 25%) | During preparation, colleagues wanted to draw lots for rest times. Interruption led to non-compliance with the manual that guides 25% solution: 6 mL/minute. Made in 43 seconds syringe with 20mL. | 6,6 | Moderate |
| **199** | [Sulfamethoxazole and trimethoprim](https://www.whocc.no/atc_ddd_index/?code=J01EE01)  (tablet 400 mg plus 80 mg) | Scheduled for 8 a.m. and administered at 9:12 a.m. | 6,8 | Moderate |
| **40** | Codeine 30mg + Paracetamol 500mg (tablet) | Pain was prescribed, but it was systematic. | 6,9 | Moderate |
| **171** | Lactate Ringer's KCl; NaCl; Glucose; MgSO4 (solution for injection) | Assistent brushed the equipment after introducing electrolytes and had a loss of volume of the drug that could not be specified. 32mL of medication was added, but the total volume after loss is not known. Flow programmed according to the prescription. | 7 | Severe |
| **172** | Lactate Ringer's KCl; NaCl; Glucose; MgSO4 (solution for injection) | Assistent brushed the equipment after introducing electrolytes and had a loss of volume of the drug that could not be specified. 32mL of medication was added, but the total volume after loss is not known. Flow programmed according to the prescription. | 7 | Severe |
| **173** | Lactate Ringer's KCl; NaCl; Glucose; MgSO4 (solution for injection) | The pump was programmed considering a total volume of 500mL in the bag, and in preparation it was aspirated with the same syringe for each electrolyte 12.5mL KCl + 7mL NaCl + 10mL glucose + 2.5mL MgSO4 and added in a vial 500mL of Lactate Ringer. In addition, there were interruptions. | 7 | Severe |
| **42** | Codeine (30 mg tablet) | Pain was prescribed, but medication was systematically aprazado apraza. Professional suffered interruptions, in addition to being in double 24 hours, responsible for three beds, being census of the day 17/23. | 7 | Severe |
| **182** | Tramadol (solution for injection, 100 mg/2 ml) | Prescribed "in case of abdominal pain". Systematic and administered in 30 minutes. | 7 | Severe |
| **10** | Azathioprine (tablet 50 mg)) | Prescribed 150mg (03 tablets), made 50 mg (01 tablet). Technician suffered interruption. | 7 | Severe |
| **117** | NPH insulin (suspension for injection) | Medication was not administered. Confirmed with patient that the same was not done. HGT = 126. | 7,1 | Severe |
| **118** | NPH insulin (suspension for injection) | Technique recorded in the prescription that did not administer medication because patient had risk of hypoglycemia. HGT (8 p.m.). Observer reports: I did not see omission being discussed with nurse on duty, being this technique also nurse. | 7,1 | Severe |
| **30** | Ceftriaxone (for injection 1g) | Manual recommends IV infusion between 15-30 minutes, done in about 85 minutes (60 drops/minute). Drug infused together with clindamycin and manual reports to be incompatible for infusion in Y. | 7,1 | Severe |
| **29** | Cefepime (for injection 1g) | This drug should be diluted in 10 ml of water or 0.9% SF and infusion between 15-30 minutes. Diluted in 100 ml of 0.9% SF and infused at 60 drops/minute, about 33 minutes. | 7,1 | Severe |
| **24** | Cefazoline (for injection 1g) | Dilution manual recommends time of direct intravenous administration between 3-5 minutes and does not recommend diluting in 0.9% saline. Done in 56 seconds and reconstituted in 10ml of 0.9 DES (aspirated all contents). | 7,1 | Severe |
| **25** | Cefazoline (for injection 1g) | Dilution manual guides direct intravenous administration between 3-5 minutes and does not recommend diluting in 0.9% saline. Reconstitution was performed in 20 ml of 0.9% saline. Administration was done in 30 seconds. | 7,1 | Severe |
| **198** | [Sulfamethoxazole and trimethoprim](https://www.whocc.no/atc_ddd_index/?code=J01EE01)  (tablet 400 mg plus 80 mg) | Prescribed 3 tablets and administered 1 tablet. | 7,1 | Severe |
| **105** | Hydralazine (tablet 25 mg) | Note in the prescription not to make antihypertensive drugs on the days of hemodialysis (Tuesday, Thursday and Saturday). Administration was made on Thursday. | 7,7 | Severe |
| **106** | Hydralazine (tablet 25 mg) | Observation in the prescription not to do antihypertensive drugs on hemodialysis days (Tuesday, Thursday and Saturday). Administration was made on Thursday. | 7,7 | Severe |
| **107** | Hydralazine (tablet 25 mg) | Note in the prescription not to make antihypertensive drugs on the days of hemodialysis (Tuesday, Thursday and Saturday). Administration was made on Thursday. | 7,7 | Severe |
| **88** | Warfarin (tablet 5 mg) | The tablet was offered to the patient, however the drug was prescribed to another patient. | 7,7 | Severe |
